# A Worldwide Study of the Relationship Between Gini Coefficients and Suicide Rates

**DOI:** 10.3390/ijerph22071110

**Published:** 2025-07-15

**Authors:** Juncheng Lyu, Jie Zhang, Dorian A. Lamis

**Affiliations:** 1 School of Public Health, Shandong Second Medical University, Weifang 261053, China; lvjuncheng79@163.com; 2 College of Public Health, Shandong University, Jinan 250012, China; 3 Department of Sociology, SUNY Buffalo State University, Buffalo, NY 14222, USA; 4 School of Medicine, Emory University, Atlanta, GA 30322, USA; dorian.lamis@emory.edu

**Keywords:** Gini coefficient, suicide rate, WHO, strain theory of suicide, relative deprivation

## Abstract

*Background:* The Gini coefficient measures how much the distribution of income or consumption within an economy deviates from an equal distribution. However, there has been a paucity of research examining the association between Gini coefficients and suicide rates in the countries of the world. *Objective:* To prove the hypothesis that the higher the Gini coefficient, the larger the relative deprivation and the higher the suicide rate, and further to verify the effect of relative deprivation on suicidality. *Methods:* Suicide rates for different countries were obtained from the World Health Organization (WHO) website. The Gini coefficients for the countries were taken from the World Bank website. Correlations were performed between the suicide rates and the Gini coefficients. SPSS 21.0 was used to analyze the data. *Results:* Overall the suicide rates and Gini coefficients decreased slightly from 2017 to 2019. There was an overall negative association between Gini coefficients and suicide rates in the countries studied. However, a different trend was observed in lower-income countries, where higher Gini coefficients were associated with higher suicide rates. The correlation between Gini coefficients and the suicide rates of females was larger than males in low- and high-income countries. However, the correlation for males was larger than females in lower-middle- and upper-middle-income countries. *Conclusions:* Current data show that Gini coefficients are negatively associated with suicide rates, but there is a different trend for lower-income countries. Economic development and the strain theory of suicide can be applied to explain the effects of relative deprivation on suicidality.

## 1. Introduction

Suicide is a serious public health problem globally, with more than 800,000 people dying by suicide each year [1]. Although the age-specific global suicide rate declined significantly between 1990 and 2019 (from 13.8 to 9.8 per 100,000 population), the total number of deaths by suicide increased by 19,897 (from 738,799 to 758,696) over the same period [2]. Income inequality is directly related to the mental health of the population [3]. Adalberto Campo-Arias and Edwin Herazo found that the correlation between inequality and suicide rates was positive and statistically significant (r = 0.70; *p* < 0.001), and that there is a positive association between economic inequality and suicide rate in Colombia [3]. Several other studies have verified that economic growth and increasing income [4,5] may contribute to the reduction of suicide rates and should be considered in suicide-prevention efforts. However, the past 20 years have seen dramatic rises in suicide rates in the United States and other countries around the world [6]. Tran and colleagues (2020) found that income inequality was positively associated with suicide rates; however, the effect on suicide rates may vary across counties [7]. Padmanathan and associates (2020) revealed that higher levels of income inequality and GDP are risk factors for youth suicide in high-income countries [8]. The Gini coefficient measures the extent to which the distribution of income or consumption among individuals or households within an economy deviates from an equal distribution; this coefficient is then used to measure the income equality of the population [9]. Lee and associates (2014) studied the correlation between economics and violent death rates in forty countries and reported that violent deaths were associated with an increase in the Gini coefficient [10]. Veisani and associates (2019) found that there was a positive inequality in Gini coefficients related to suicide deaths in Iran [11]. Sugita and Izuno examined the relationship between increases in the Gini coefficient and the mortality rate among young adults in Japan and predicted that there was a positive association between Gini coefficients and the suicide rate of young adults. MH Kazemi-Galougahi announced that lower income might be considered a risk factor for completed suicide [12]. Another study from the literature indicated that unemployment rate (*p* ≤ 0.001), Gini coefficient (*p* ≤ 0.001), and population growth rate (*p* ≤ 0.001) had a positive relationship with the suicide rate [13]. However, Hsu CY et al. showed that suicide rates were associated with indicators of socioeconomic deprivation (a population with non-professional jobs and low median household income) and social fragmentation (measured by the proportion of unmarried adults and divorced/separated adults), but not with the Gini coefficient [14].

With regard to the association between economic status and suicide rate, some studies have probed the correlation between them. Nicolas Raschke considered that low income and financial difficulties were risk factors for suicidality [15], and O. Claveria found that economic growth may lead to suicidality [16]. A recent systematic review indicated that lower income levels were more likely to exhibit a higher suicide risk [17]. O. Claveria et al. found that decreases in economic uncertainty have a greater impact on suicide mortality than increases [18]. The strain theory of suicide and mental disorders is based on theoretical frameworks established by previous sociologists for criminal behaviors [19,20,21]. There are four sources of the strain theory of suicide: (1) differential value strain, (2) the discrepancy between aspiration and reality, (3) relative deprivation, and (4) the lack of coping skills [22]. In a previous study [23], we verified that relative deprivation is an important risk factor for suicide as explained in the strain theory of suicide. The previous literature has verified that when psychological strain exceeds the tolerance threshold, it would be either released inward [24] or released outward [25]. The Gini coefficient is an important index to evaluate the economic relative deprivation factor of suicide in the world. Previous studies have only examined the relationship between the Gini coefficient and suicide rates based on limited areas or countries. There has been no literature reporting on the relationship between Gini coefficients and suicide rates from a worldwide perspective. The objective of the current study was to explore whether the Gini coefficient is associated with suicide rates and verify whether relative deprivation strain contributes to suicidality. This study analyzed the relationship between Gini coefficients and suicide rates based on a world database. The research hypothesis was that the higher the Gini coefficient in a country, the larger the relative deprivation, and therefore, the higher the suicide rates.

## 2. Materials and Methods

### 2.1. Data Collection

The suicide-rate data from the WHO mainly come from official reports of member countries and are integrated and verified through the global mortality database. The main sources include Civil Registration and Vital Statistics Systems (CRVS), censuses and surveys, police records and autopsy reports, disease surveillance points, etc., and suicide deaths have specific codes (X60-X84, Y87.0). The WHO first implements the data sorting and cleaning process (including formatting and checking for completeness and internal consistency, whether the data conform to the ICD codes for suicide death, checking for obvious errors or outliers, and filling in missing data) and then establishes the statistical model to estimate and calculate the suicide rate.

Due to the different age structures in various countries, the age-standardized suicide rate is used to eliminate the bias caused by different age compositions. The purpose of the “age-standardized suicide rate” is to eliminate the influence of age-structure differences among different populations, so as to ensure more fair and comparable comparisons of suicide rates in different countries. Age-standardized suicide rates can be calculated by dividing the expected number of deaths in this age group under the standard population composition by the total standard population.

The age-standardized suicide rates (per 100,000 population) of 183 countries from 2017 to 2019 were obtained from the World Health Organization (WHO) website [1]. The Gini coefficients, GDP, and other economic indexes of the countries were obtained from the World Bank database [9].

### 2.2. Variable Explanation and Data Integration

The Gini coefficient was invented in 1912 by the Italian statistician and demographer Corrado Gini [26], and it is the most commonly used inequality index. Graphically, the Gini index is represented by the surface area between a line at 45° and a Lorenz curve (a graphical representation of the cumulative percentage of the total income versus the cumulative percentage of the population that receives that income, where income increases left to right) [26]: area A in the following equation divided by the total surface area under the line (A + B), that is, IG = A/(A + B) [26].

The Gini coefficient measures the extent to which the distribution of income or consumption among individuals or households within an economy deviates from an equal distribution [9]. A Gini coefficient of 0 represents perfect equality, while a 1 indicates perfect inequality. Lower than 0.2 means absolute average income, 0.2–0.3 means relatively average, 0.3–0.4 means relatively reasonable, 0.4–0.5 indicates a large income gap, and above 0.5 indicates a wide income gap.

The classification of national income levels by the World Health Organization (WHO) directly adopts the official standards of the World Bank. This classification of national income levels is based on Gross National Income per capita data (GNI per capita). Per capita GNI = Gross National Income (GNI)/population in the middle of the year, and it is updated once a year (usually on 1 July) to define low-income, lower-middle-income, upper-middle-income, and high-income countries/economies. More information on the country classification can be found on the WB website [27].

National income is categorized into four levels and is recorded as follows: 1 = low income, 2 = lower-middle income, 3 = upper-middle income, and 4 = high income.

Microsoft Office software (Excel software 2010 version) and the merge method were used to integrate the data from the different databases by the same field (Country Code) so as to link the Gini coefficient and suicide rate country by country.

### 2.3. Ethical Approval

The data come from the WHO and the World Bank open database. This study was approved by the Ethics Committee of Shandong Second Medical University (Approval Code: 2021YX084). The databases were obtained through a reasonable application.

### 2.4. Statistical Analysis

The $\bar{x}\pm s$ and median (quartile range, Q) were used to describe the quantitative data and n (%) for qualitative variables, respectively. The Spearman correlation method was used to explore the relationship between Gini coefficients and suicide rates. *p* < 0.05 was set for the significance level, and SPSS 21.0 was used to analyze the data.

## 3. Results

### 3.1. Description of the Sample

This study indicates that the suicide rates of 183 countries decreased slightly from 10.43 ± 9.37 to 10.08 ± 8.70 (per 100,000 population). The male suicide rate decreased from 16.74 ± 16.28 to 16.13 ± 14.96 (per 100,000 population), and the female suicide rate decreased from 4.66 ± 3.69 to 4.53 ± 3.59 (per 100,000 population) from 2017 to 2019. However, the median of both the overall suicide rate and male suicide rate initially showed a decrease but then demonstrated an increasing trend over the following three years.

The mean of the Gini coefficient decreased from 35.53% to 35.42% from 2017 to 2019, with a decreasing and then an increasing trend. In addition, the median of the Gini coefficient decreased from 35.35% to 34.50%, as shown in Table 1.

### 3.2. The Relationship Between Gini Coefficients and Suicide Rates

The Spearman correlation method was used to examine the relationship between suicide rates and Gini coefficients. The results indicate that there were negative statistically significant correlations between Gini coefficients and suicide rates, not only for the overall suicide rate, but also for male and female suicide rates when analyzed separately. However, no significant correlation was found between the Gini coefficient and the gender ratio (male/female) of suicide rates, as shown in Table 2.

### 3.3. Relationship of Gini Coefficient on Suicide Rate in Different National Income Groups

There were 12.96% low-income, 25.00% lower-middle income, 25.00% upper-middle income, and 37.04% high-income countries. In order to reduce the confounding bias of different income levels, we combined the 3 years of data to investigate the relationship between Gini coefficients and suicide rates in different national income groups.

In low-income countries, there was a significant negative correlation between Gini coefficients and female suicide rates. Regarding both the overall suicide rate and the male suicide rate, the correlation coefficient was positive but did not reach significance.

For the lower-middle-income countries, the correlation coefficient was negative, but only the male suicide rate was significant (*p* < 0.05).

In the upper-middle-income countries, there was a significant negative correlation between Gini coefficients and suicide rates, not only for the overall suicide rate, but also for male and female suicide rates (*p* < 0.05).

In the high-income countries, there was a negative correlation (*p* < 0.05) found between the Gini coefficient and the overall suicide rate and the female suicide rate.

With regards to the countries in total, the Gini coefficient had a negative statistically significant correlation with both the overall suicide rate, the male suicide rate, and the female suicide rate, respectively (*p* < 0.05). The results are shown in Table 3.

When we compared the gender difference in different national income groups, we found that the degree of correlation between the Gini coefficient and the suicide rates of females was larger than with males in the low- and high-income countries. However, the degree of correlation among males was larger than for females in the lower-middle and upper-middle income countries.

## 4. Discussion

In this study, we found that the means of the overall suicide rate and the male and female suicide rates all showed a slightly decreased trend, whereas the median of the overall suicide rate and male suicide rate evidenced a decreasing and then increasing trend from 2017 to 2019 globally. Given that suicide rates usually follow a skewed distribution, the change trend of the median more accurately reflects the trend in suicide rates, and the increasing trend in 2019 should be further explored to explain this change.

The Gini coefficient, as an index to measure the extent of income equality, is also considered an indicator of the level of social redistribution of wealth. Theoretically, a high Gini coefficient reflects inequality of income, with a low level of distribution of wealth and high relative deprivation, and it is likely to lead to a high suicide rate, although some previous research has found a positive association between Gini coefficients and suicide rates in some countries [10,11] and specific populations [28].

We found negative correlations globally between the Gini coefficient and suicide rates, not only in the vertical dimension (different years) but also in the horizontal dimension (different national income levels). The potential explanations for the inconsistent results are as follows: First, the Gini coefficient is an index of the extent of income equality, but it only partly reflects the economic level of a country. Furthermore, suicide behaviors are complex, and there are many potential confounding variables, such as social environment and cultural variables, which are more salient than the Gini coefficient. Second, the correlation between the Gini coefficient and suicide rates is inconsistent; it is different across various regions and countries. The correlation may be positive for specific regions and countries but negative for the world.

Although in low-income countries, there was a positive correlation trend between Gini coefficients and both the overall suicide rate and the male suicide rate, this is not statistically significant. This positive correlation trend is similar to that reported in the previous literature [10,11,28] and confirms the research hypothesis that the larger the Gini coefficient in a country, the higher the rates of suicide. The reason may be that in the low-income countries, economic or income variables play a more important role in suicide behaviors. Larger Gini coefficients reflect a lower economic level and unequal social wealth redistribution. In environments where wealth is unequally distributed, people may be more likely to perceive the income gap and relative deprivation. The perceived relative deprivation is one source of psychological strain [29]. Consequently, when the relative deprivation or psychological strain exceeds the tolerance threshold, it would be either released inward (such as in suicidal behavior and suicidal ideation) [24] or released outward (such as in criminal behavior) [25]. As to females residing in low-income countries, there was a negative correlation between the Gini coefficient and suicide rates. One possible explanation for this finding is that females are mainly responsible for household work in low-income countries, which may contribute to perceptions of income gap and relative deprivation of income for females being less than that of males. At the same time, there may be other social or cultural variables that contribute more to female suicides than does the Gini coefficient.

The current study highlights the association between the Gini coefficient and suicide rates. However, more research is needed to examine this relationship, particularly by comparing different countries. Whether the Gini coefficient is a strong predictive index for suicide rate or not needs further study, and different kinds of countries may have different results. In low-income countries, the Gini coefficient is a stronger predictor of suicide than in middle- and high-income countries. Moreover, although the Gini coefficient may be a predictive index for suicide rates, there are many more potential variables that should be considered in future investigations, such as social factors, cultural factors, value factors, cognition factors, etc.

## 5. Conclusions

There is a lack of research about the association between Gini coefficients and suicide rates worldwide, and the previous literature reveals inconsistent conclusions. Despite the following limitations and biased data, it is essential to explore the association between them. The current study verifies that Gini coefficients are associated with suicide rates, specifically that Gini coefficients are negatively associated with suicide rates, but with a different trend for low-income countries. Generally speaking, this study validates the research hypothesis that the higher the Gini coefficient, the larger the relative deprivation, and therefore the higher the suicide rates. An underdeveloped economy and the effects of relative deprivation contribute to suicidality. The concept of relative deprivation and the strain theory of suicide can be applied to explain the higher suicide rates. The Gini coefficient has a certain level of meaningful reference in identifying the high-risk countries for suicide, and it can be of great significance in suicide management and prevention.

## 6. Limitations

The current study only included data from available open-access databases, and 3 years of data may not be sufficient to reflect changes in long-term trends. Without controlling for other potential variables, results should be considered in light of this limitation. Given that the databases used were from the WHO and the World Bank, the quality of the databases is also a potential source of research bias.

## Figures and Tables

**Table 1:** Description of the suicide rates and Gini coefficients from 2017 to 2019.

Variables	N	$\bar{x}\pm s/$M(Q)		
		2017	2018	2019
Overall suicide rate *	183	10.43 ± 9.37 8.65 (7.33)	10.24 ± 9.04 8.19 (7.33)	10.08 ± 8.70 8.28 (7.14)
Male suicide rate *	183	16.74 ± 16.28 13.59 (13.18)	16.43 ± 15.69 13.33 (12.43)	16.13 ± 14.96 13.35 (12.51)
Female suicide rate *	183	4.66 ± 3.69 4.08 (4.06)	4.58 ± 3.59 3.85 (3.81)	4.53 ± 3.59 3.79 (3.84)
Gender ratio (M/F)	183	3.80 ± 1.87 3.44 (1.98)	3.78 ± 1.94 3.37 (1.87)	3.78 ± 1.86 3.36 (2.03)
Gini coefficient (%)	76/84/59	35.53 ± 6.99 35.35 (10.75)	35.76 ± 6.88 35.30 (10.18)	35.42 ± 7.56 34.50 (12.20)

Note: * indicates the unit is /100,000.

**Table 2:** The relationship between the Gini coefficient and the suicide rate.

Variables	Spearman Coefficient (r_s_) with Suicide Rate (/100,000)		
	2017	2018	2019
Overall suicide rate (/100,000)	−0.335 ** p = 0.003	−0.337 ** p = 0.002	−0.382 ** p = 0.003
Male suicide rate (/100,000)	−0.341 ** p = 0.003	−0.319 ** p = 0.003	−0.340 ** p = 0.009
Female suicide rate (/100,000)	−0.236 * p = 0.041	−0.272 * p = 0.012	−0.351 ** p = 0.007
Gender ratio (male/female)	−0.095 *p* = 0.420	0.003 *p* = 0.978	−0.030 *p* = 0.822

Note: * and ** indicate that the correlation was significant at 0.05 and 0.01, respectively.

**Table 3:** Relationship between the Gini coefficient and suicide rates in different national income groups.

National Income Group	Spearman Coefficient (rs) of Gini Coefficient on Suicide Rate (/100,000)		
	Overall Suicide Rate	Male Suicide Rate	Female Suicide Rate
Low-income	0.191 *p* = 0.287	0.473 *p* = 0.071	−0.573 * p = 0.033
Lower-middle-income	−0.236 *p* = 0.066	−0.258 * p = 0.050	−0.128 *p* = 0.210
Upper-middle-income	−0.365 ** p = 0.001	−0.334 ** p = 0.003	−0.270 * p = 0.013
High-income	−0.178 * p = 0.041	−0.159 *p* = 0.061	−0.208 * p = 0.021
All countries	−0.352 ** p < 0.001	−0.335 ** p < 0.001	−0.280 ** p < 0.001

Note: * and ** indicate that the correlation was significant at 0.05 and 0.01, respectively.

## Data Availability

The data come from the WHO and the WB open data and can still be obtained from the corresponding author for reasonable reasons.
